# A new delivery model to increase adherence to methadone maintenance treatment

**DOI:** 10.22088/cjim.9.1.104

**Published:** 2018

**Authors:** Saeid Komasi, Mozhgan Saeidi, Payam Sariaslani, Ali Soroush

**Affiliations:** 1 Clinical Research Development Center, Imam Reza Hospital, Kermanshah University of Medical Sciences, Kermanshah, Iran.; 2 Cardiac Rehabilitation Center, Imam Ali Hospital, Kermanshah University of Medical Sciences, Kermanshah, Iran.; 3 Neurology Department, Imam Reza Hospital, Kermanshah University of Medical Sciences, Kermanshah, Iran; 4 Lifestyle Modification Research Center, Imam Reza Hospital, Kermanshah University of Medical Sciences, Kermanshah, Iran

**Keywords:** Methadone maintenance treatment, Substance abuse, Technology

**Dear Editor,**

Today, a large number of Iranian addicts are treated in methadone maintenance treatment (MMT) centers (1). According to the current procedure, each patient has treatment record in only one clinic. So, each patient is able to get prescribed medications and other medical services of the center. Receiving drugs and center-based services has always faced serious challenges such as access (2). For example, some patients with mobility jobs, people with movement restrictions and physical conditions, and patients living in rural and remote areas are unable to refer to get the medication according to a regular timetable (3). The mentioned issues are a serious challenge for persistence and adherence to MMT. Under such circumstances, a significant proportion of patients abandoned treatment plan and they lost the ancillary services, including medical visits, psychological interventions, and physical and emotional support from a social worker. Ultimately, this situation led to an increased the risk of relapse and resume high risk behaviors related to substance abuse (4). Social damage and financial losses of the country's health system are only some of the negative consequences of this situation.

Based on these considerations, presentation of the strategies and constructive recommendations in support of the related organizations is a necessity. Despite the several offers raised, it seems that the abolition of the system receiving drugs from the origin center and the use of digital identification systems can be useful and practical (5). In the form of drug delivery system, patients can receive their daily dosages in any of the center across the country. This project is done in such a manner that each patient first enrolls in an MMT center and fills out a medical record form. Then, the doctor will determine the types of medication and drug dose based on the patients’ medication history. In the first three months of treatment (i.e. until the stabilization of the dose), visits or appointments will be face to face. After the period and stabilized drug dose, the patient who receives medication is no longer confined to the origin center. This means that each patient is issued an entity identifier such as a smart card for medication. By issuing the smart card, the patient can receive quota set medication use in any part of the country. Definitely to prevent abuse of patients, it is recommended that the patient's identity be verified through the finger, eye, or facial recognition digital sensors (5). Meanwhile, bringing up an instruction can be useful based on a mandatory visit to the center of origin for medical examination and psychological services at least once a month.

So far, few studies have been conducted to review and confirm the delivery format of pharmaceutical services (6, 7). However, previous studies show that the facial assessment via computer evaluation and photo anthropometric variations in facial features are standard references for personal identification in the field of health and forensic (8, 9). Thus, our offer could possibly be effective in increasing adherence to treatment and reducing problems caused by the access to origin MMT center. As a result, we recommend using this pilot model for at least a one-year period. Then, if the benefit of this model is in practice, this proposal could be implemented permanently.

## Funding:

No source of funding was used.

## Conflict of Interest:

None of the authors have conflicts of interest to report.
